# EQ-5D visual analog scale and utility index values in individuals with diabetes and at risk for diabetes: Findings from the Study to Help Improve Early evaluation and management of risk factors Leading to Diabetes (SHIELD)

**DOI:** 10.1186/1477-7525-6-18

**Published:** 2008-02-27

**Authors:** Susan Grandy, Kathleen M Fox

**Affiliations:** 1 Health Economics and Outcomes Research, AstraZeneca Pharmaceuticals LP, Wilmington, DE, USA; 2 Strategic Healthcare Solutions, LLC, Monkton, MD, USA

## Abstract

**Background:**

The EQ-5D was used to compare burden experienced by respondents with diabetes and those at risk for diabetes.

**Methods:**

A survey including the EQ-5D was mailed to individuals with self-reported diabetes, as well as those without diabetes but with the following risk factors (RFs): (1) abdominal obesity, (2) body mass index ≥ 28 kg/m^2^, (3) dyslipidemia, (4) hypertension, and (5) cardiovascular disease. Non-diabetes respondents were combined into 0–2 RFs and 3–5 RFs. Mean EQ-5D scores were compared across groups using analysis of variance. Multivariable linear regression modeling identified factors affecting respondents' EQ-5D scores.

**Results:**

Complete responses were available from >75% of each cohort. Mean EQ-5D index scores were significantly lower for respondents with type 2 diabetes and 3–5 RFs (0.778 and 0.792, respectively) than for those with 0–2 RFs (0.870, *p *< 0.001 for each); score for respondents with type 2 diabetes was also significantly lower than for those with 3–5 RFs (*p *< 0.001). Similar patterns were seen for visual analog scale (VAS). For both VAS and index scores, after adjusting for other characteristics, respondents reported decreasing EQ-5D scores as status moved from low to high risk (-6.49 for VAS score and -0.045 for index score) to a diagnosis of type 2 diabetes (-9.75 for VAS score and -0.054 for index score; *p *< 0.001 vs. 0–2 RFs for all).

**Conclusion:**

High-risk and type 2 diabetes groups had similar EQ-5D scores, and both were substantially lower than in low-risk respondents.

## Introduction

It has been estimated that diabetes mellitus affects approximately 21 million people in the U.S. [1]. Complications from diabetes include blindness, kidney disease, nerve damage, arterial disease, abnormal cholesterol levels, hypertension, heart disease, and stroke. Heart disease and stroke account for 65% of deaths in patients with diabetes, with a death rate 2–4 times higher than in adults without diabetes [2]. Diabetes is the fifth leading cause of mortality in the U.S., and is associated with increasing economic burden, estimated at $132 billion in 2002, up from $98 billion in 1997 [3].

Diabetes and its complications and comorbidities substantially affect patients' health-related quality of life (HRQoL) [4-7]. The impact of treatment, complications, and comorbidities has been documented to adversely affect HRQoL among individuals with type 2 diabetes mellitus [8]. Yet, there is little information on HRQoL among individuals who do not have diabetes but are at risk for diabetes. While several disease-specific instruments have been used to measure the HRQoL of patients with diabetes, there is a need for generic HRQoL measures as well, to allow comparisons with populations without diabetes. In particular, such measures can be used to compare the incremental burden experienced by patients with diabetes and those without diabetes but with similar comorbidities and risk factors.

A frequently used generic HRQoL instrument is the EuroQoL EQ-5D [9]. The objective of this investigation was to compare EQ-5D scores of individuals diagnosed with diabetes and those with varying levels of cardiometabolic risk, using data from the Study to Help Improve Early evaluation and management of risk factors Leading to Diabetes (SHIELD). This investigation will ascertain whether the burden of having risk factors for diabetes impacts HRQoL in a similar way as having diabetes. SHIELD is a 5-year longitudinal survey-based study that is being conducted to better understand the overall burden of illness of people living with diabetes as well as those at risk for its development.

## Methods

A 12-item general population screening questionnaire was used to identify individuals with a diagnosis of diabetes and those with risk factors associated with a diagnosis of diabetes. In 2004, the screening survey was mailed to a stratified random sample of 200,000 U.S. households [10]. This was followed by a baseline survey in which a sample of identified cases were followed up with a more detailed survey assessing each individual's health status, health knowledge and attitudes, and current health-related behaviors and treatments. A total of 22,001 baseline survey questionnaires were mailed in late 2004. Respondents freely volunteered to complete the survey without enticement, and no IRB approval was required.

### Risk factors

In addition to self-reported diagnosis of diabetes, responses to the screening questionnaire were used to identify respondents with the following risk factors: (1) abdominal obesity (waist circumference: men >97 cm, women >89 cm), (2) body mass index (BMI) ≥ 28 kg/m^2^, (3) dyslipidemia (reported diagnosis of cholesterol problems of any type), (4) hypertension (reported diagnosis of high blood pressure), and (5) history of cardiovascular disease (reported heart disease/myocardial infarction, narrow or blocked arteries, stroke, coronary artery bypass graft surgery, angioplasty, stents, and/or surgery to clear arteries). These risk factors were derived from the literature, national guidelines, and expert opinion as modifiable or treatable risk factors for the future development and/or diagnosis of diabetes [11,12]. Respondents with 0–2 risk factors were classified as low risk and those with 3–5 risk factors were grouped as high risk for a diagnosis of diabetes. This paper will focus on respondents with type 2 diabetes, low risk (0–2 risk factors), and high risk (3–5 risk factors).

### EQ-5D

The EQ-5D was used as a measure of respondents' HRQoL and utility values. The EQ-5D provides a simple descriptive profile and a single index value for health status [9,13]. The EQ-5D self-reported questionnaire includes a visual analog scale (VAS), which records the respondent's self-rated health status on a graduated (0–100) scale, with higher scores for higher HRQoL. It also includes the EQ-5D descriptive system, which comprises 5 dimensions of health: mobility, self-care, usual activities, pain/discomfort, and anxiety/depression. The VAS provides a direct valuation of the respondent's current state of health, whereas the descriptive system can be used as a health profile or converted into an index score representing a von Neumann-Morgenstern utility value for current health [9]. The level of problem reported on each of the EQ-5D dimensions determines a unique health state. Health states are converted into a weighted health state index by applying scores from the EQ-5D preference weights elicited from general population samples. These weights lie on a scale on which full health has a value of 1 and dead a value of 0. For this study, U.S. population weights were used to convert to an EQ-5D index score [14].

### Statistical analysis

For each group (type 2 diabetes, high risk and low risk), the mean EQ-5D scores both overall and by dimension are reported. Statistical comparisons across groups (with emphasis on comparisons between the type 2 diabetes group and the other groups) were performed using analysis of variance with Fisher's least significant difference post-hoc testing, with *p *< 0.01 considered significant.

In addition, multivariable linear regression modeling was used to identify those factors that most affected respondents' EQ-5D scores, including the diabetes risk group (type 2 diabetes, high risk or low risk). Even though the EQ-5D is a 5-item scale, linear regression modeling has been used in previous HRQoL studies. These investigations have demonstrated the comparability of EQ-5D with other generic HRQoL instruments and its usefulness in identifying determinants of health states [15-17]. The following sociodemographic factors were included: age, gender, race, geographic region, household income and size, BMI category, and group status (low risk, high risk, or type 2 diabetes) to determine if diabetes risk was independently associated with HRQoL after adjusting for the sociodemographic characteristics as well as assessing if the sociodemographic factors were independently associated with HRQoL. The sociodemographic categories are those used by the U.S. Census Bureau to describe the U.S. population and are utilized in SHIELD to demonstrate the representativeness of the study sample. Reference categories were selected as the largest group except for income (highest category) and diabetes risk status (type 2 diabetes). Using the methodology of Cavrini and associates and Sitoh and colleagues [18,19], an ordinal variable for the EQ-5D index was created by categorizing the continuous variable into 4 levels, and an ordered logit regression model was used to confirm the multivariate linear regression. Results were similar between the linear and ordered regressions, so the linear regression results were presented since this statistical technique is more widely used.

## Results

Of the 22,001 baseline survey questionnaires mailed, 17,640 were returned (response rate: 80.2%). Complete responses for the EQ-5D were available from >75% of each cohort (5,639 of 7,403 for low risk, 5,370 of 6,742 for high risk, and 3,849 of 5,000 for type 2 diabetes). The sociodemographic characteristics of the baseline respondents who completed the EQ-5D in each group are shown in Table 1. The low- and high-risk groups had a significantly greater proportion of respondents who were younger, white, and had more education and higher income compared with the type 2 diabetes group, p < 0.01.

**Table 1:** Characteristics of SHIELD baseline respondents who completed the EQ-5D, by group

**Characteristics**	**Low Risk n = 5,639**	**High Risk n = 5,370**	**Type 2 Diabetes n = 3,849**
Age, mean, yrs (SD)	47.0 (16.4)*	58.9 (14.6)*	60.3 (13.1)
Women, %	65.5%*	56.6%	57.8%
Race, % white	88.3%*	88.4%*	85.0%
Education, % with some college or higher	74.0%*	67.3%*	63.9%
Income, % with <$40,000/year	36.5%*	46.3%*	52.5%
Geographic region, %			
Northeast	18.8%	19.7%	19.9%
South	34.2%	36.7%	38.5%
Midwest	25.5%	25.5%	23.5%
West	21.4%	18.1%	18.0%

* *p *< 0.01 for comparison with type 2 diabetes

### VAS state of health

**Figure 1:**
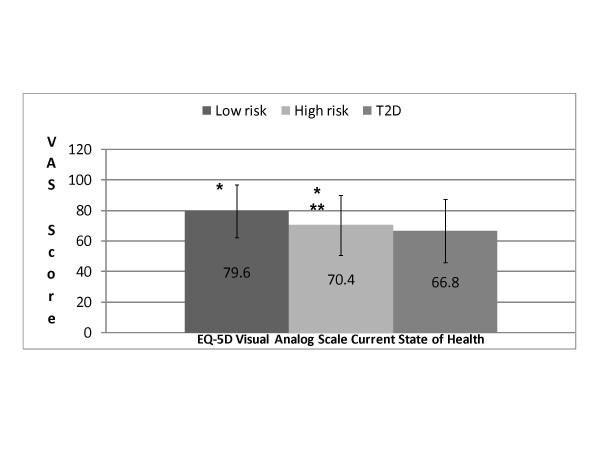
**Mean EQ-5D VAS scores by group**. *p < 0.001, low risk versus T2D and low risk versus high risk. **p < 0.001, high risk versus T2D. EQ-5D = EuroQoL- 5 Dimensions; T2D = type 2 diabetes.

Mean EQ-5D VAS scores were significantly higher for low- and high-risk respondents (79.6 and 70.4, respectively) compared with type 2 diabetes respondents (66.8, *p *< 0.001 for each) (Figure 1). In addition, the mean VAS score for low-risk respondents was significantly higher than the mean score for the high-risk group (*p *< 0.001). A greater proportion (34.5%) of respondents at low risk for diabetes rated their current state of health >90 on the VAS, compared with respondents with type 2 diabetes (13.9%) or at high risk for diabetes (17.7%).

### Utility index scores

**Figure 2:**
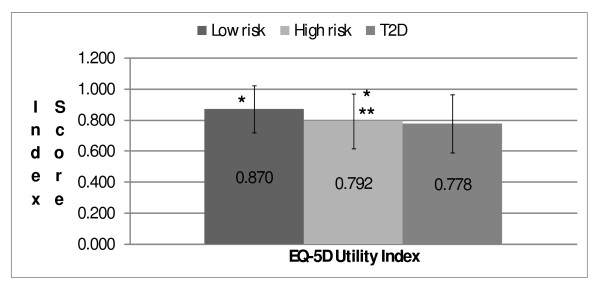
**Mean EQ-5D utility index scores by group**. *p < 0.001, low risk versus T2D and low risk versus high risk. **p < 0.001, high risk versus T2D. EQ-5D = EuroQoL- 5 Dimensions; T2D = type 2 diabetes.

The pattern of EQ-5D utility index scores was similar to that observed for VAS scores (Figure 2). Mean EQ-5D index scores were significantly higher for low- and high-risk respondents (0.870 and 0.792, respectively) than for those with type 2 diabetes (0.778, *p *< 0.001 for each). The mean index score for low-risk respondents was significantly higher than the mean for the high-risk group (*p *< 0.001).

### EQ-5D dimensions

Examination of each of the 5 dimensions of the EQ-5D showed similar rating scores for the type 2 diabetes and high-risk groups, with both groups more likely to report more difficulties or limitations compared with the low-risk group (Table 2). A much higher proportion of respondents with type 2 diabetes (47.9%) and those at high risk (43.4%) reported having mobility problems compared with those at low risk (17.1%) (*p *< 0.001 for both) (Table 2). Percentages of respondents reporting problems with self-care were generally low across all groups; however, respondents with type 2 diabetes (8.5%) or at high risk (6.5%) were more likely to report this problem compared with those at low risk (2.7%). More than twice as many respondents with type 2 diabetes (36.1%) and those at high risk (33.3%) reported having problems performing usual activities compared with those at low risk (15.7%) (*p *< 0.001). More respondents with type 2 diabetes (61.1%) and at high risk (61.8%) reported experiencing some pain or discomfort compared with those at low risk (43.5%) (*p *< 0.001). Additionally, a greater proportion of those with type 2 diabetes (10.5%) and those at high risk (9.4%) reported extreme pain or discomfort compared with low-risk respondents (4.2%) (*p *< 0.001). The proportion of respondents reporting moderate levels of anxiety or depression was similar across respondents with type 2 diabetes (26.1%) and at high risk (24.9%), and lowest in respondents at low risk for diabetes (19.9%).

**Table 2:** Proportion of respondents reporting problems on each EQ-5D dimension in the baseline SHIELD survey, by group

**Proportion of respondents reporting some or unable, or moderately/extremely, %**	**Low risk**	**High risk**	**Type 2 Diabetes**
**Decreased mobility**	17.1*^	43.4*	47.9
**Difficulty with self-care**	2.7*^	6.5*	8.5
**Problems performing usual activities**	15.7*^	33.3*	36.1
**Pain or discomfort**	43.5*^	61.8	61.1
**Anxious or depressed**	19.9*^	24.9	26.1

EQ-5D = EuroQoL-5 Dimensions; *p < 0.001 for comparison with type 2 diabetes; ^ *p *< 0.0001 for comparison of high risk to low risk

### Multivariable linear regression models

Diabetes risk status was significantly associated with HRQoL after adjusting for sociodemographic factors (Table 3). Compared with type 2 diabetes respondents, the low-risk respondents (9.02 for VAS score and 0.049 for index score; p < 0.0001) and high-risk respondents (3.18 for VAS score and 0.009 for index score; *p *= 0.008) reported higher EQ-5D scores. The model F statistic was 94.0 for VAS score and 83.6 for index score, and the model r-square was 0.16 for VAS score and 0.15 for index score.

**Table 3:** Multivariable linear regression analyses of factors impacting EQ-5D scores in SHIELD baseline respondents*

**Variables**		**EQ-5D VAS score n = 14,383**		**EQ-5D index score n = 14,378**	
		
		**Beta coefficient**	**SE**	**Beta coefficient**	**SE**
Diabetes risk group					
	Low risk	9.02†	0.45	0.049†	0.004
	High risk	3.18†	0.39	0.009†	0.004
	Type 2 diabetes	(reference)		(reference)	
Age (yrs)					
	18–24	5.69†	0.94	0.052†	0.008
	25–34	0.39	0.63	0.011	0.006
	35–44	(reference)		(reference)	
	45–54	- 0.65	0.51	- 0.025†	0.005
	55–64	- 0.34	0.54	- 0.033†	0.005
	65–74	2.82†	0.58	- 0.011†	0.005
	≥75	- 0.45	0.64	- 0.031†	0.006
Gender					
	Female	- 1.16†	0.32	- 0.029†	0.003
	Male	(reference)		(reference)	
Race					
	White	(reference)		(reference)	
	Black	1.20†	0.59	0.014†	0.005
	Other	- 1.66	0.96	- 0.015	0.009
Household Income ($) per year					
	<22,500	- 13.03†	0.49	- 0.121†	0.004
	22,500–39,999	- 6.68†	0.49	- 0.066†	0.004
	40,000–59,999	- 3.62†	0.50	- 0.037†	0.004
	60,000–89,999	- 1.71†	0.49	- 0.020†	0.004
	≥90,000	(reference)		(reference)	
Geographic region					
	Northeast	2.70†	0.80	0.029†	0.007
	Middle Atlantic	2.34†	0.58	0.026†	0.005
	East North Central	2.28†	0.56	0.021†	0.005
	West North Central	2.58†	0.70	0.023†	0.006
	South Atlantic	1.87†	0.54	0.015†	0.005
	East South Central	0.42	0.73	- 0.002	0.007
	West South Central	1.75†	0.63	0.014†	0.006
	Mountain	1.03	0.74	0.011	0.007
	Pacific	(reference)		(reference)	
Household size (no. of members)					
	1	(reference)		(reference)	
	2	- 0.77	0.43	- 0.009†	0.004
	3	- 1.68†	0.53	- 0.018†	0.005
	4	- 1.05	0.59	- 0.010†	0.005
	≥5	- 2.52†	0.64	- 0.022†	0.006
Body mass index (kg/m^2^) group					
	Underweight	- 3.12†	1.43	- 0.017	0.013
	Normal weight	(reference)		(reference)	
	Overweight	- 1.33†	0.46	- 0.007	0.004
	Obese	- 6.57†	0.47	- 0.047†	0.004

*Scores indicate change from reference group. †*p *< 0.05 versus reference group

EQ-5D = EuroQoL-5 Dimensions; VAS = visual analog scale; SE = standard error

Other sociodemographic characteristics were significantly associated with EQ-5D scores upon adjusting for diabetes risk status, including age, income, obesity, gender, race, geographic region, and household size (Table 3). Increasing age was associated with decreased quality of life for EQ-5D index scores, although not for VAS scores. Respondents aged 55–64 years or 75 years and older reported the greatest negative impact on quality of life (*p *< 0.001 vs. respondents aged 35–44 years), with those aged 18–24 years having the highest EQ-5D scores. The analysis of VAS scores for current health state showed no clear trend across age groups compared with respondents aged 35–44 years. For both VAS and index scores, respondents' HRQoL decreased as household incomes decreased; those with incomes <$22,500 reported the greatest negative impact on HRQoL (*p *< 0.001 vs. ≥$90,000 in both models).

For both EQ-5D scores, obesity (BMI ≥ 28 kg/m^2^) was associated with significantly lower HRQoL (p < 0.0001), while black race was associated with significantly higher HRQoL compared with white race (p < 0.05) (Table 3). The results for other sociodemographic factors indicate that female gender and household size of 3 or ≥5 were associated with a negative impact on EQ-5D VAS scores, and female gender and a household size ≥2 were associated with a negative impact on EQ-5D index scores. HRQoL was significantly higher among residents of other geographic regions compared with the Pacific region for both EQ-5D scores.

## Discussion

The EQ-5D results from the SHIELD survey demonstrate that respondents at low risk for the development and diagnosis of diabetes experienced the lowest proportion of self-reported difficulties in all 5 measured dimensions (mobility, self-care, usual activities, pain/discomfort, and anxiety/depression) compared with respondents with type 2 diabetes or at high cardiometabolic risk. Overall EQ-5D scores, whether measured by VAS or index score, were substantially higher in the low-risk group compared with the high-risk and type 2 diabetes groups, even after adjusting for sociodemographic characteristics. The high-risk and type 2 diabetes groups had similar health profiles and overall scores, although the latter reported somewhat lower overall HRQoL.

Respondents with type 2 diabetes reported the highest rates of difficulties with mobility, self-care, and performing usual activities. Similar proportions (> 60%) of respondents with type 2 diabetes and at high risk for diabetes reported experiencing some pain or discomfort. Reported rates of moderate anxiety or depression were also similar for respondents with type 2 diabetes and those at high risk. These findings were similar to other studies, which found impaired physical and social functioning as measured by the SF-36 among individuals with type 2 diabetes [20,21].

This study provides evidence of the HRQoL of respondents at risk for diabetes as well as those with type 2 diabetes using a generic HRQoL instrument. The EQ-5D in the present study allowed for comparisons of respondents not yet diagnosed with diabetes since the dimensions were relevant to overall well-being. Other studies have typically compared type 2 diabetes patients with the general population [20-22]. Studies using the Medical Expenditure Panel survey (MEPS) examined individual risk factors and a cluster of similar cardiometabolic risk factors (BMI ≥25 or ≥30 kg/m^2^, hyperlipidemia, hypertension and diabetes) as used in the present study and found a similar significant deleterious impact on HRQoL as measured by the EQ-5D and SF-36 [22,24].

Construct validity of the EQ-5D has been established in several chronic diseases, including rheumatoid arthritis [25,26], stroke [27], and AIDS [28]. However, it has not been widely used in diabetes studies, where preference is to use the various disease-specific HRQoL instruments. Yet, the EQ-5D is a valid measure of HRQoL with modest correlation with measures of impairment (e.g., joint scores, HIV scales) and high correlation with patients' perception of their disabilities (e.g., Health Assessment Questionnaire, Barthel Index, and Modified Rankin scale) [25,27,28]. The EQ-5D has performed equally well when compared with other generic HRQoL and utility-based instruments, including the Health Utilities Index Mark 2 and 3 and SF-6D [26,29].

In the present study, no clear trend in the EQ-5D VAS scores across age groups was observed, even though there was a strong age association in the EQ-5D index score. In rheumatoid arthritis, Hurst and colleagues [25] found a negative association with age for both the utility and VAS scores; yet Hart and colleagues [17] found no age association among patients with type 1 diabetes mellitus. It is unclear in the present study why current health status (VAS) was reported as better in 65-74-year-old respondents compared with 35-44-year-old respondents.

The EQ-5D utility scores from this study provide a preference-based score that can be used to calculate quality-adjusted life years for future cost-effectiveness analyses of treatment or prevention of diabetes and evaluating healthcare interventions both clinically and economically. Since SHIELD respondents are representative of the U.S. population with or at risk for diabetes, the EQ-5D utility scores would be useful for national and multi-national comparisons for quality-adjusted life-year assessments.

The present study provides evidence of the impact of type 2 diabetes and high risk on HRQoL in a large sample with a high survey response rate. Moreover, the respondents are representative of the U.S. population, and the evaluation of HRQoL was done using a standardized, validated measure so that norm-based results are provided. However, it should be noted that household panels such as those used for this survey tend to under-represent the very wealthy and very poor segments of the population, and do not include military or institutionalized individuals. In addition, SHIELD relied only on self-reported data to identify samples of respondents, without clinical or laboratory confirmation. These limitations are the same for most survey-based methodologies.

## Conclusion

The EQ-5D results from the SHIELD survey show that respondents with type 2 diabetes and those at high risk for future diagnosis of diabetes report decreased overall HRQoL and more difficulty with mobility, self-care, and usual activities compared with those at lower risk. Reported reductions in HRQoL may be due to related comorbidities or to overall health burden. Reducing cardiometabolic risk factors may lead to significant improvements in HRQoL even before diabetes is diagnosed in high-risk respondents. Respondents with a low risk for diabetes consistently reported the lowest rates of problems or difficulties across all 5 health dimensions measured by the EQ-5D. Further follow-up is needed to track HRQoL profiles over time, as those who are at risk for diabetes are diagnosed and learn to cope with their disease.

## Abbreviations

BMI – Body mass index; EQ-5D – EuroQoL-5 Dimensions; HRQoL – Health-related quality of life; RF – Risk factor; SHIELD – Study to Help Improve Early evaluation and management of risk factors Leading to Diabetes; U.S. – United States; VAS – Visual analog scale

## Competing interests

SHIELD, the SHIELD Study Group, and the preparation of this manuscript were supported by funding from AstraZeneca LP. Dr. Susan Grandy is an employee of AstraZeneca LP, and Dr. Fox is a research consultant for AstraZeneca LP.

## Authors' contributions

SG participated in the conception, design and coordination of the SHIELD study and helped to draft the manuscript. KF performed the statistical analysis and drafted the manuscript. All authors read and approved the final manuscript.
